# Warming the head of hypothermic patient – is it always safe?

**DOI:** 10.1186/s13049-016-0337-x

**Published:** 2016-12-03

**Authors:** Paweł Podsiadło, Tomasz Darocha, Sylweriusz Kosiński

**Affiliations:** 1 Polish Society for Mountain Medicine and Rescue, ul. Dębowa 2, Szczyrk, 43-370 Poland; 2 Severe Hypothermia Treatment Center, Krakow, Poland; 3 Department of Anaesthesiology and Intensive Care, John Paul II Hospital, Jagiellonian University Medical College, Krakow, Poland; 4 Department of Anaesthesiology and Intensive Care, Pulmonary Hospital, Zakopane, Poland; 5 Tatra Mountain Rescue Service, Zakopane, Poland

**Keywords:** Accidental hypothermia, Rewarming, Prehospital treatment

## Abstract

The head warming in hypothermic victims is an alternative way of heat donation, which does not inhibit shivering and does not impede the access to the patient’s chest. It seems to be a safe method in mild hypothermia. The authors of the review article “Accidental hypothermia – an update” suggest this way of heat donation, without indicating precisely, in which group of patients it can be applied. In severe hypothermia, the brain-protective effect of cold is well known. The decreased need of oxygen allows good neurological outcome after long lasting cardiac arrest. Therefore, in deep hypothermia, the brain tissue should be rather insulated from the heat source than warmed.

## Main text

The publication “Accidental hypothermia – an update” is a valuable, comprehensive review of current knowledge of hypothermic patients’ management, especially in prehospital settings [1]. It contains clear and useful guidelines, which are very important in the mountain rescue.

The authors touch on the very interesting topic of heat donation by head warming. In the chapter “Prehospital insulation, rewarming, rescue collapse and afterdrop” this method is mentioned as equal to other ways of heat donation. It is based on the experimental study that has demonstrated good effectiveness and the avoidance of shivering inhibition [2]. The study, however, was performed on mildly hypothermic patients. In mild hypothermia, due to the adrenergic stimulation, cardiac output and respiratory drive are increased [3] and therefore the risk of ischaemic brain damage is low. For this reason the brain-protective effect of low temperature does not play an essential role in mild hypothermia, so the head warming seems to be safe for these patients.

In moderate and severe hypothermia, the cardiac output and ventilation are decreased, accordingly to reduced metabolic demands. Severe hypothermia can especially predispose to cardiac instability and cardiac arrest can easily occur. Decreased brain temperature and thus reduced need of oxygen, allows to avoid the ischaemic damage [4]. Thanks to the protective effect of low temperature, full neurological recovery is possible, even after cardiac arrest lasting several hours [5].

The animal study in deep hypothermia has demonstrated that fast brain tissue warming (preceding the core temperature increase) can lead to brain ischaemia [6]. For this reason, keeping the low gradient between a brain temperature and a core temperature during rewarming, can be essential for neuronal survival. Sometimes, shielding the head from the heat source can be beneficial, which is mentioned by the authors in further part of their paper [1].

In our opinion, the heat donation by warming the head should be limited strictly to mildly hypothermic victims.
